# An accurate method of radiological assessment of acetabular volume and orientation in computed tomography spatial reconstruction

**DOI:** 10.1186/s12891-015-0503-8

**Published:** 2015-02-25

**Authors:** Marek Jóźwiak, Michał Rychlik, Bartosz Musielak, Brian Po-Jung Chen, Maciej Idzior, Andrzej Grzegorzewski

**Affiliations:** Department of Paediatric Orthopaedics and Traumatology, Poznan University of Medical Sciences, ul. Fredry 10, 61-701 Poznan, Poland; Division of Virtual Engineering, Poznan University of Technology, ul. Piotrowo 3, 60-965 Poznan, Poland; Department of Orthopaedics and Paediatric Orthopaedics, Medical University of Lodz, ul. Kościuszki 4, Łódź, Poland

**Keywords:** Acetabulum, Computed tomography, Three-dimensional reconstruction, Hip dysplasia, Spastic hip disease

## Abstract

**Background:**

Two-dimensional (2D) measurements of acetabular morphology and orientation are well known; there is less information on these acetabular characteristics in three dimensions. One important reason is the lack of standardized reference planes for the pelvis, especially in relation to the spinopelvic unit; another is that no method precisely assesses the acetabulum in three-dimensional (3D) orientation based on its axis rather than on the directions of the edges of the acetabular rim. We present an objective, highly reliable and accurate, axis-based approach to acetabular anthropometry in the measurement of acetabular volume and spatial orientation in both normal and pathologic hips. This was done using reference planes based on the sacral base (SB) and true acetabular axis in 3D computed tomography (CT) pelvic reconstruction.

**Methods:**

Radiological examinations of 30 physiologic pelves (60 acetabula) were included in the study. Reliability and accuracy of the method were verified by comparing acetabular angles in 2D pelvic scans with 3D reconstructions. We also applied the method to two pathologic acetabula.

**Results:**

Comparison of axis position in the horizontal plane revealed significant positive correlations between 2D angle measurements (acetabular anteversion angle [AAA] and anterior acetabular index [AAI]) and 3D measurement of anteversion angle (p < 0.001 and p = 0.012, respectively). In the frontal plane, there was no difference between abduction angle, measured on topogram, and inclination angle, obtained from a 3D model (p = 0.517). In the sagittal plane, there was a significant negative correlation between AAA and acetabular tilt (p < 0.001). Inter- and intra-observer reproducibility was excellent for determination of the sacral-base plane and assessment of volume, with Fleiss κ coefficients of 0.850 and 0.783, respectively, and intraclass correlation coefficients of 0.900 and 0.950, respectively. Inter-observer reproducibility for evaluation of acetabular axis ranged from 0.783 to 0.883, and intra-rater reliability ranged from 0.850 to 0.900 for all 3D angles.

**Conclusions:**

Our method is a new, reliable diagnostic tool for assessing the acetabula in both normal and pathologic hip joints. The sacral-base plane can be used as a stable reference that takes the relationship of the acetabulum to the spinopelvic unit into consideration.

**Electronic supplementary material:**

The online version of this article (doi:10.1186/s12891-015-0503-8) contains supplementary material, which is available to authorized users.

## Background

Hip acetabular dysplasia is a general term that refers to spatial deformations of the acetabulum, in terms of abnormal depth, volume, and spatial orientation of its components in relation to other pelvic elements or the proximal end of the femur [1-5]. Radiological evaluation of the acetabula of the hip joint is important for assessing the progression of hip joint pathology, such as spastic hip joint instability observed in children with cerebral palsy [6,7], or for determining the extent of acetabular wall deficit in developmental dysplasia of the hip.

In chronic hip diseases, anthropometric measurements of acetabular geometry and orientation can determine the decision-making process regarding surgical treatment and affect outcome [8-10]. The method of treatment is usually chosen on the basis of two-dimensional (2D) or three-dimensional (3D) imaging examinations. Improvements in 3D imaging such as magnetic resonance (MRI) and computed tomography (CT) have given clinicians the opportunity to find practical tools for qualitative and quantitative evaluation of acetabular pathology [11,12]. Acetabular characteristics examined in two dimensions are well known, but there is still relatively little knowledge of acetabular characteristics examined in three dimensions, especially in diseases like developmental dysplasia of the hip or spastic hip disease [5,13-15], and the use of 2D imaging in preoperative planning and outcome prognosis in spatial, 3D joint deformities is insufficient. Although there have been studies of volume assessment under both physiologic and pathologic conditions using 3D images, there are too few methods for evaluation of volume under pathologic conditions, such as spastic hip, in pediatric patients [8,9,15-17].

Acetabular orientation is currently evaluated using 3D imaging, but it is based on either subjective methods, or objective methods of low accuracy [5,15,18-21]. To our knowledge, no study has accurately assessed the position of the acetabulum in true spatial reconstructions of the pelvis in spastic hip disease [15]. One reason for this is the lack of standardized, stable reference planes of the pelvis and an independent measurement method [18-20]. Pelvic reference planes used today tend to focus on pelvic tilt but do not take into account the very important global morphologic element of the spinopelvic unit, represented by pelvic incidence [22-26].

Even as 3D techniques have become more popular, the lack of a stable method of acetabular measurement continues. A new, objective, axis-based approach to acetabular anthropometry is required. Thus, the main objectives of this paper are to: (1) present a new method for measuring acetabular volume and spatial orientation in both normal and pathologic hip joints using new reference planes and the acetabular axis in 3D-CT pelvic reconstructions; (2) present a highly reliable and accurate measurement method, and (3) present its diagnostic applications to hip joint pathologies, including extremely dysplastic acetabula with completely reversed orientation.

## Methods

### Study design

In this prospective study, CT scans of consecutive patients with surgical, not orthopedic, diagnoses in 2012–2014 were examined. No patient had pelvic bone pathology. All measurements were performed electronically using rapid-prototyping and computer-aided design software (Rhinoceros, Robert McNeel & Associates, Seattle, WA, USA; ScanIP, Simpleware, Exeter, UK) to acquire 3D representations of our sample.

### Ethical approval

Approval No. 499/10 was provided by the Poznan University of Medical Sciences Bioethical committee. All the participants (adults) gave a written consent for participation in the study.

### Method of measurement

The method of measurement consisted of four stages: generation of a computer model of the pelvis, determination of reference planes, determination of acetabular volume, and determination of the acetabular axis relative to the plane of reference.

Rapid-prototyping and CAD software were used to acquire 3D representations of our sample, as follows:

### Generation of a 3D computer model of the pelvis

**Figure 1:**
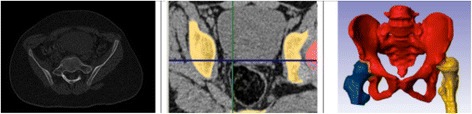
**The segmentation process in generating the 3D model of the pelvis.**

Pelvic models were generated using classic CT data obtained with the GE LightSpeed VCT 64-slice CT system (GE Healthcare, Little Chalfont, UK). Slice thickness of the accepted scans was <1.5 mm (0.63 or 1.25 mm). Scans formatted as DICOM files were transferred to ScanIP for processing of 3D images. The areas of the pelvic bones were marked on each scan. Based on this, the ScanIP algorithm generated a triangle-surface mesh describing the geometry of the analyzed pelvis. The accuracy of this process (segmentation) is crucial in obtaining quality 3D models (Figure 1). The resulting 3D model was subsequently exported to Rhinoceros, a specialized design software for measuring spatial images.

### Determination of reference planes

**Figure 2:**
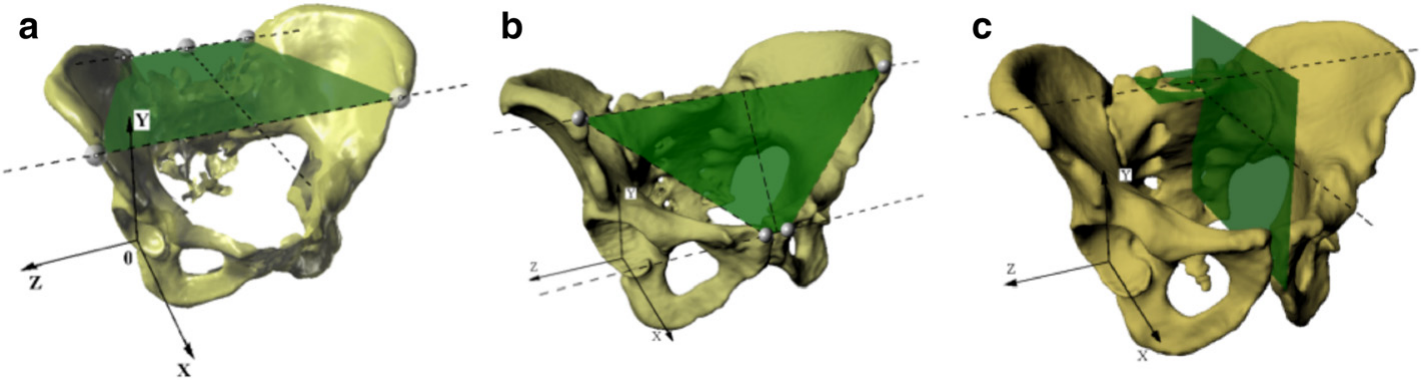
**Planes of reference for measurement and pelvic XYZ coordinate system. (a)** Recommended by the STC; **(b)** anterior pelvic plane; and **(c)** the sacral-base plane, recommended by the authors of this research; each picture with visualization of pelvic XYZ coordinate system. 2D, two-dimensional; 3D, three-dimensional; STC, Standardization and Terminology Committee of the International Society of Biomechanics.

Three sets of reference planes were considered: the plane recommended by the Standardization and Terminology Committee of the International Society of Biomechanics (STC plane) (Figure 2a) [27]; the anterior pelvic plane (APP) described by Robinson and Lewinnek (Figure 2b) [15,18-21,28-30]; and planes established with the use of the sacral base (SB) (Figure 2c). The APP is a commonly used reference plane for the assessment of acetabular cup orientation after total hip replacement [31]. However, there is no consensus as to the reliability of this method for acetabular orientation [18-20,22]. The STC plane is part of the joint coordinate system and is set as a reference plane for reporting hip joint motion [21,27].

The reference planes adopted in the present study are established by the sacral base (SB, Figure 2c). The horizontal plane of reference is defined as a plane interpolated from the mesh points located on the surface of the SB (the basic assumption was to have at least 30 points; however, we used a mesh with a density of 3 × 3 mm that always gives no less than 100 points and usually over 150 points). The vertical plane is perpendicular to the horizontal one and coincides with the geometric center of the SB (which is automatically set based on the previously applied mesh of points) and the midpoint of a line connecting the centers of the pubic tubercles, which were set based on a minimum of 30 points (mesh of points set on those surfaces at a density of 1 × 1 mm, usually yielding about 100 points) marked on each pubic tubercle surface. This method of plane selection led to the determination of the pelvic XYZ coordinate system (Figure 2c) with the following components: 00: the pelvic origin point (0,0,0) coincident with the geometric center of the SB; X0: the axis formed by the intersection of the sagittal and horizontal planes, pointing anteriorly; Z0: the axis orthogonal to the X0 axis and lying on the horizontal plane, pointing to the right or left depending from the hip joint being evaluated; and Y0: the axis perpendicular to both the X0 and Z0 axes, pointing cranially.

### Determination of acetabular volume

**Figure 3:**
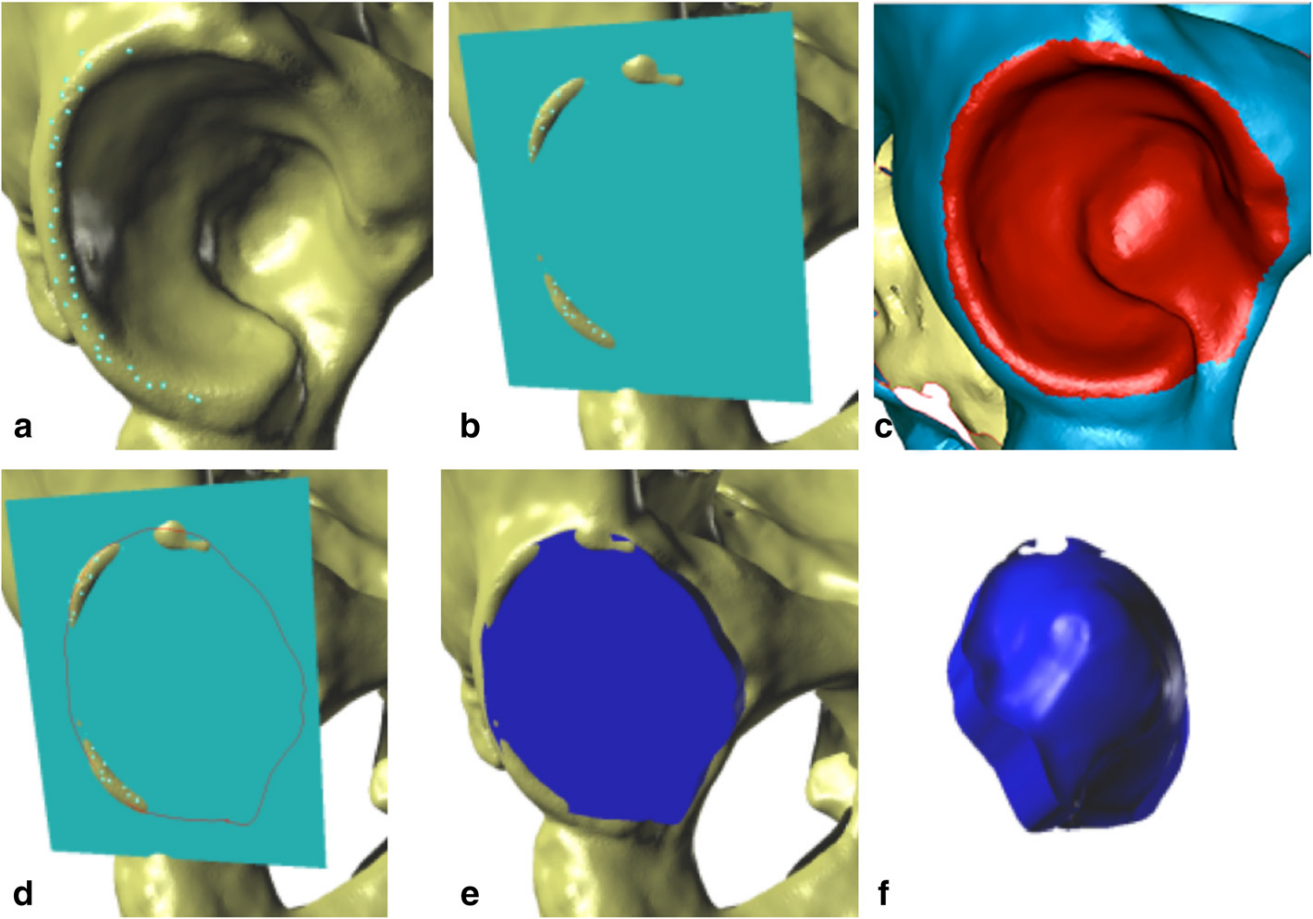
**Acetabular volume measurements. (a)** Points set on the acetabular rim; **(b)** acetabular-opening plane created as an interpolated plane based on previously set rim points; **(c)** area of the acetabulum, marked in red; border curve, the border between the red and blue surfaces; **(d)** projection of the border curve on the acetabular-opening plane; **(e)** determination of the top surface of the acetabular space; **(f)** visualization of the solid figure representing the acetabular volume.

In the proposed method, volume is measured using the acetabular-opening plane, established by the acetabular rim, which limits the area of the hip socket. Volume determination consisted of the following steps: Step 1, finding the acetabular-opening plane: This plane is an interpolated plane based on the points (at least 30) situated on the acetabular rim (Figures 3a, b); Step 2: circumscribing the surface of the acetabulum and the border curve: The area of the acetabulum includes the lunate surface, the acetabular fossa, and the acetabular notch and is limited by the acetabular rim; the border curve is a line corresponding to the acetabular rim, based on the points previously set on the rim (Figure 3c); Step 3, projection of the border curve onto the acetabular-opening plane (Figure 3d); Step 4, determining the top surface of the acetabular space, the surface sectioned off from the acetabular-opening plane with the projection of the border curve (Figure 3e); Step 5: determining the surfaces contained in the acetabular space and measuring the final volume; these surfaces are perpendicular to the acetabular-opening plane, directed towards the bottom of the acetabulum and limited by the surface marked in step 2. From these steps, a solid figure is obtained (Figure 3f), which subsequently is measured.

To summarize, the acetabular volume measured by this method is the space confined by the surfaces of the acetabulum and the acetabular-opening plane externally and enclosed on the sides by the perpendicular surface joining the curve projected onto the opening with the surface of the acetabulum (Figure 3f).

### Determination of the acetabular axis relative to the reference planes

**Figure 4:**
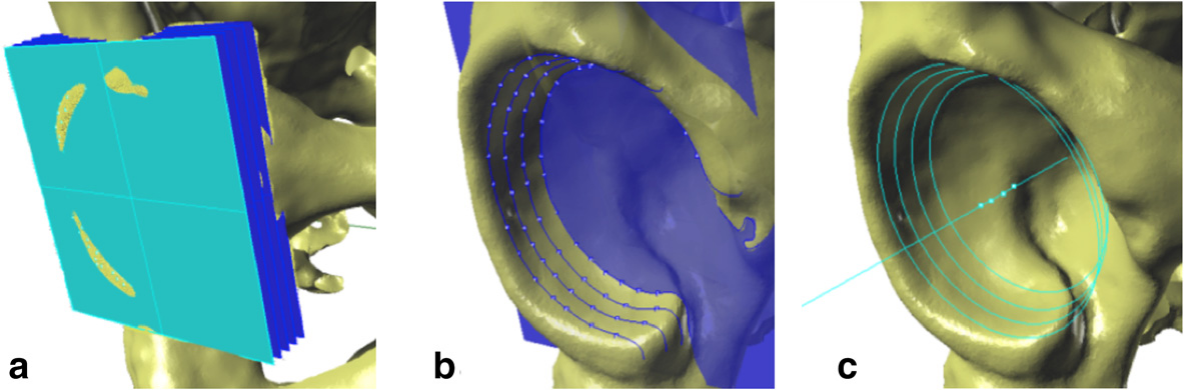
**Steps to determining the acetabular axis: (a) Generating a set of section planes (parallel to the acetabular-opening plane); (b) finding the curve that is the intersection of section planes with acetabular surface, and subsequent marking of points over the curve; (c) fitting the circles to the intersection points and establishing the acetabular axis by finding an average trend line that joins the centers of the circles.**

The acetabular axis is defined as the line joining the centers of the circles fitted to the edges determined by the intersection of the lunate surface with the planes parallel to the acetabular-opening plane. The procedure for determining the acetabular axis is as follows: step 1, determining a set of section planes: drawing planes parallel to the acetabular-opening plane, at a distance of 1 mm each, to the top of the acetabulum (Figure 4a); step 2, drawing base curves and points: finding the curve that is the intersection of the lunate surface with reference planes; marking points (at least 30) on each curve (Figure 4b); step 3, fitting circles to the points on curves: using points set on each curve, a circle is fitted to the selected points on each section and its center marked (Figure 4c); step 4, determining the acetabular axis: the axis is established by interpolation the trend line over the centers of the circles (Figure 4c).

**Figure 5:**
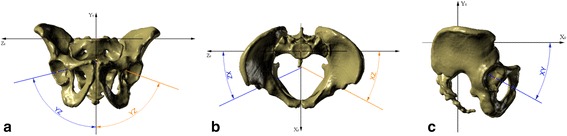
**Angles of three-dimensional orientation of the acetabulum. (a)** Inclination angle, **(b)** anteversion angle, and **(c)** tilt angle.

Based on estimation of the axis, the orientation of the acetabulum is described using three angles (Figure 5): inclination angle (measured in frontal plane): the angle created by the y-axis and the acetabular axis; anteversion angle (measured in horizontal plane): the angle created by the z-axis and the acetabular axis; and tilt angle (measured in the sagittal plane): the angle created by the x-axis and the acetabular axis.

### Method validation

Our method was verified in several ways. Measurement error was first assessed by means of a test measurement performed on the polyethylene acetabular cup of a hip joint implant. The test was performed using three techniques: analytical calculations (mathematical model based on the technical documentation), measurement of the volume of a 3D CT reconstruction model, and a computer model obtained from a 3D structural light scanner.

**Figure 6:**
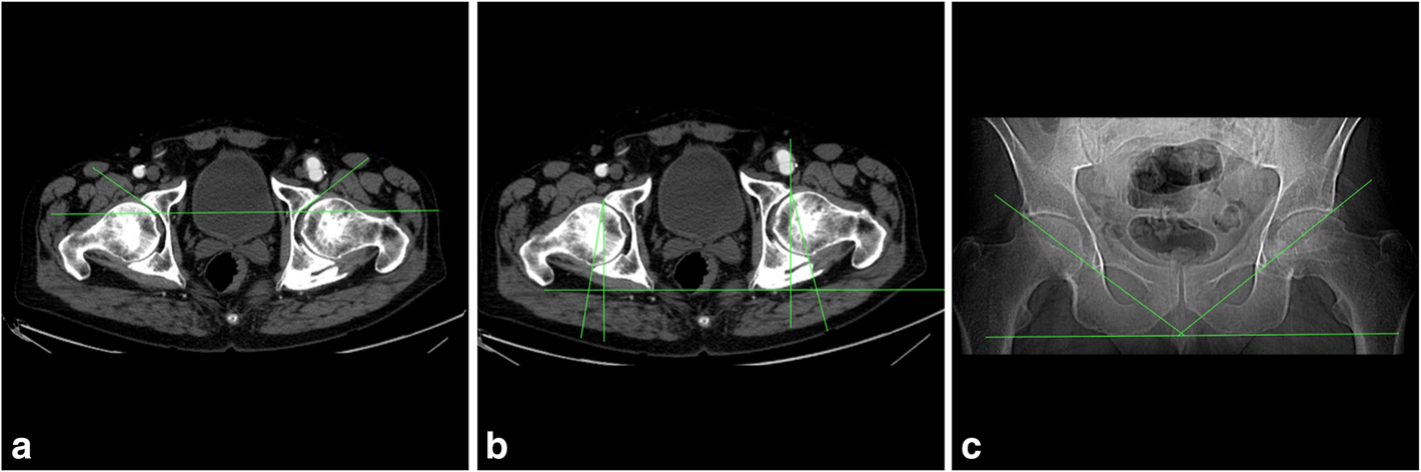
**Angles measured using two-dimensional technique: (a) Anterior acetabular index, (b) acetabular anteversion angle, and (c) abduction angle based on the topogram.**

Next, a comparison between our method and classic 2D measurement was made to authenticate the new technique. Thirty adult pelvic CT scans (28 males, 2 females; 60 acetabula) were included in the study. Records were taken consecutively from patients with surgical, non-orthopedic diagnoses who were free of any pelvic bone lesions. The 30 CT scans were assessed using the following 2D angles: anterior acetabular index (AAI) (Figure 6a) and acetabular anteversion angle (AAA) (Figure 6b) in the horizontal plane [32]; and abduction angle in the frontal plane (based on the topogram taken at the beginning of the CT-scanning process) (Figure 6c) [33], the angle between the line connecting upper lateral and lower medial rims of the acetabulum and the line connecting the lower edge of the ischial tuberosities. Abduction angle was measured in 25 cases, as topograms were not recorded for three male and two female patients. A 3D model of the pelvis was then generated based on the scans previously obtained, and all measurements were performed.

Finally, our method was assessed for reliability. Ten 3D pelvic reconstructions randomly chosen from this research were assessed twice by three investigators (one advanced, two novices) at minimum 2-week intervals. Evaluation of SB reference plane, acetabular axis, and volume were done for comparison.

### Statistical analysis

The linear dependence of the 2D and 3D angles in the horizontal plane, as well as in the two remaining planes, frontal and sagittal, was examined with Pearson’s product–moment correlation. Correlations ≠ 0 with a p-value < 0.05 were considered statistically significant. The Fleiss κ coefficient was used to assess the inter-rater reliability. Intraclass correlation coefficients (ICCs) were used to assess intra-rater reliability.

## Results

### Volume

The volume of the polyethylene acetabular cup of the hip joint implant assessed by CT was similar to that described in the specifications for the implant (Table 1). Mean acetabular volume in patients examined for the purpose of this study was 39.80 mL (range, 19.42–60.8 mL).

**Table 1:** **Volume measurements of the polyethylene acetabular cup of the hip joint implant**

**Model type**	**Volume [mL]**
Analytical model	6.51
Model reconstructed from CT imagining	6.65
Model reconstructed from 3D scanning	6.65
Value of the measurement error	0.14

3D, three-dimensional; CT, computed tomography.

### Spatial orientation

**Figure 7:**
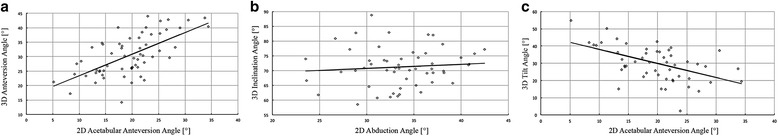
**Trend line variations based on observations of main parameters measured in 2D and 3D technique: (a) 3D anteversion angle; (b) 3D inclination angle; (c) 3D tilt angle.** 2D, two-dimensional; 3D, three-dimensional.

Comparison of the axis position in the horizontal plane revealed a significant positive correlation between angles measured on 2D images and those based on the 3D reconstructions. There was a significant correlation between AAA and 3D anteversion angle as well as between AAI and 3D anteversion angle, with Pearson correlation coefficients of 0.640 and 0.324, respectively (Table 2, Figure 7).

**Table 2:** **Pearson’s correlation coefficients for relationships between 2D and 3D acetabular measurements**

**2D measurements**	**3D measurements**		
	**Anteversion angle (** ***p*** **-value)**	**Inclination angle (** ***p*** **-value)**	**Tilt angle (** ***p*** **-value)**
**Anteversion angle**	0.640 (*p* < 0.001)	--	– 0.449 (*p* < 0.001)
**Anterior acetabular index**	0.324 (*p* = 0.012)	--	--
**Abduction angle**	--	0.090 (*p* = 0.517)	0.157 (*p* = 0.255)

2D, two-dimensional; 3D, three-dimensional.

In the frontal plane, there was no correlation between abduction angle measured on the topogram (2D) and the inclination angle obtained from the 3D model (r = 0.090; Table 2, Figure 7). We therefore assessed the accuracy of the 2D topograms by measuring pelvic tilt; i.e., measuring the distance between the upper edge of the symphysis pubis and the line connecting the centers of both femoral heads [34,35]. The average distance between those points was 1.57 cm, with a wide range of values (−1.30 cm to 4.87 cm) (Table 3) corresponding, according to Tannast at al. [34], to 23.3–92.2 degrees of pelvic tilt (Table 3).

**Table 3:** **Topograms showing the distance between the upper edge of the symphysis pubis and a line connecting the centers of both femoral heads (distance X) and corresponding pelvic tilt**

	**Distance X (cm)**	**Pelvic tilt (degrees)**
**Mean**	1.57	55.3
**Minimum**	−1.30	23.3
**Maximum**	4.87	92.2

In the sagittal plane there was significant negative correlation between AAA and measured in 3D, with a Pearson correlation coefficient of −0.449 (Table 2, Figure 7), but no correlation between 2D abduction angle and 3D tilt angle (Table 2).

Figure 7 shows trend lines of three main comparisons of 3D parameters with 2D parameters.

The average 3D parameters of acetabular orientation are presented in Table 4.

**Table 4:** **Three-dimensional parameters of acetabular orientation**

**Parameter**	**Mean**	**Range**
**Anteversion angle (degrees)**	30.37	14.25 – 43.99
**Inclination angle (degrees)**	70.59	58.52 – 82.86
**Tilt angle (degrees)**	31.43	13.82 – 54.91

### Inter- and intra-rater agreement

Inter- and intra-rater reliability for determination of SB plane and acetabular axis and volume are presented in Table 5. Fleiss κ coefficients were in favor of excellent inter-observer reproducibility, and ICC values demonstrated excellent intra-rater reliability in the determination of SB plane. Inter- and intra-rater reliability in the assessment of angles of acetabular orientation (Table 5) was also excelent.

**Table 5:** **Inter- and intra-rater reliability for determination of SB plane and acetabular axis and volume**

**Measurement**	***n***	**Inter-rater agreement -** κ **(%)**	**Intra-rater agreement - ICC (%)**	**Level of agreement**
**SB plane**	10	0.850	0.900	excellent
**3D anteversion angle**	20	0.783	0.850	excellent
**3D inclination angle**	20	0.833	0.850	excellent
**3D tilt angle**	20	0.883	0.900	excellent
**Acetabular volume**	20	0.783	0.950	excellent

3D, three-dimensional; ICC, intra-class correlation coefficient; κ, Fleiss kappa coefficient; SB, sacral base.

## Discussion

MRI and CT scanning have become standard hip joint imaging techniques, but precise anthropometric measurement methods are still being developed. With the introduction of 3D-image diagnostic tools, the evaluation of acetabular geometry has become more independent of the position of the pelvis during imaging [35-39]. This is important because the lack of standard pelvic positioning during imaging can lead to significant discrepancies in the results of 2D image parameters. For example, anterior pelvic tilt can completely change the section plane, causing the lower part of the acetabulum to look like the posterior wall and making determination of ante- or retroversion of the acetabulum difficult [11,35-39]. This type of measurement error, similar in some ways to the parallax phenomenon, is completely eliminated in the 3D technique presented here.

There have been studies that attempted to establish anthropometric parameters of the normal and dysplastic acetabulum based on 3D images; however, subjective, descriptive methods were mainly used for acetabular orientation, especially in patients with dysplastic or spastic changes. These studies, which focused on the orientation of components in total hip replacement, could provide objective descriptions of the position of the acetabular component in relation to the pelvis, but the descriptions were based on reference planes of low accuracy in dynamic situations because of changes in pelvic tilt with different body positions [5,15,18-20,40,41]. APP and STC planes are surfaces determined by anatomic points such as anterior superior iliac spine (ASIS) or posterior superior iliac spine (PSIS). However, the ASIS and PSIS are anatomic structures of uneven area that cannot be represented by geometric points, which may also reduce the accuracy of these planes. Thus, the APP and STC plane are inadequate for precise assessment of acetabular orientation.

The problem of a pelvic reference plane is now even more important in light of the discussion of pelvic incidence as the most important parameter describing the position of the pelvis in relation to the spine [22-26]. The relationship of the sacrum and SB to the hip joint is constant and independent of body position, making the SB a very stable anatomic landmark [26] and the basic plane of reference in the present method.

There are two other reasons for using the SB plane as a reference. The first is the lack of end points of the ASIS and PSIS on standard CT scans, which may occur in everyday practice. The second is the accuracy of setting the SB plane. Although determining the anatomical location of the anterior and posterior spines in healthy individuals is quite straightforward, the location of the points that define its end is not as clear. Using the method of setting mesh of points on the base of the sacrum makes it possible to determine a unique and repeatable plane of reference. The assumed minimum of 30 points demarcating each reference plane and the acetabular-opening plane takes physiological differences into consideration. We believe this increases the repeatability and objectivity of results, which was confirmed by the excellent inter- and intra-rater agreement in determining the SB plane.

Furthermore, to establish acetabular spatial orientation, we used an objective relationship of the axis to the planes of reference. Using averaged data (i.e., the points on the acetabular rim) to establish the acetabular-opening plane reduces measurement error, which was confirmed by the excellent inter- and intra-rater agreement in determining the orientation of the acetabulum. This study uses the true axis of the acetabulum, rather than the plane of entrance (or the acetabular plane matrix) or the axis perpendicular to this plane, to assess its orientation [8,15,22,42-44]. In 3D technique, we believe it is better to use a multisectional method than simply the acetabular-opening plane as a reference. Because the acetabulum is not a perfect sphere, the acetabular-wall axis is not necessarily perpendicular to the acetabular-opening plane, which could have a substantial influence on determination of the true orientation.

Using the present method, we have determined acetabular volume and axis by focusing on the acetabular-opening plane. Different methods of volume measurement, focusing on different elements, have been reported. Some of these methods are precise and measure true geometry in a 3D environment [16]. Others are limited by inconsistent approaches to each parameter, especially when used for pathologic conditions in pediatric patients [8,11,17,43]. We have addressed a discrepancy in the definition of acetabular volume. According to some previous publications, acetabular volume is defined as the volume of a sphere that includes the arc of the acetabular roof circumference or as the depth of the acetabulum that contains the femoral head [8]. In this study, acetabular volume is determined using the area circumscribed by the acetabular-opening plane, which is adjacent to the acetabular rim.

The methodology presented here also simplifies measurement technique. It is based only on evaluation of the geometry of acetabular structures without extra-acetabular elements such as the femoral head, femoral neck, or location of the triradiate cartilage [11,12,16,45]. We believe this is the first trial of a true 3D method of volume measurement performed in patients with spastic hip [8,15,43] see the Additional file 1.

To validate the present diagnostic method, we compared 2D and 3D measurement of angles. In CT imaging, the X-rays penetrate the body perpendicular to its long axis. However, during scanning the pelvis may tilt, which can strongly influence classic anthropometric measurement of cross sections [34-39]. Only measurements based on a spatial reconstruction will reproduce the actual relationships with high accuracy.

Because of the ability of CT scanning to create axial images, the correlation between 3D anteversion angle and 2D AAI and AAA angles was significant. This indirectly confirmed the accuracy of our method. However, it was difficult to find a 2D angle in the frontal plane corresponding to 3D inclination angle. For this reason, we compared 3D inclination angle with abduction angle measured on the topogram. There was no correlation, however, because of low accuracy of the measurement made in a 2D plane. The position of the pelvis is not standardized for a topogram, which was demonstrated by the span of nearly 70 degrees of pelvic tilt in our sample and which has been reported by a number of authors [34-37,46]. The situation was improved for measurements related to the sagittal plane of the pelvis. There is no appropriate plane angle to describe acetabular tilt. Thus, 3D measurement was compared with 2D angles in the frontal and horizontal planes. While a negative correlation was observed between 3D acetabular tilt angle and 2D anteversion angle (the negative correlation was due to the different axes of these angles), there was no relationship between 3D tilt and abduction angle measured on the topogram.

### Limitations

This study has some limitations. First, it presents a new diagnostic technique focusing on healthy cases, with only case presentation of its application to pathological hips (see Appendix). Second, as it presents a new method, our results could be compared only with 2D methods, which do not provide measurements in at least three planes. Third, this method is dedicated to high-resolution, 3D CT reconstructions, which expose patients to high levels of radiation. In the future, this method may be adapted to pelvic MRI.

## Conclusions

The present method is new measurement tool for the acetabulum that may be valuable in both normal and dysplastic hip joints. The SB plane can be used as a stable reference that takes the relationship of the acetabulum to the spinopelvic unit into consideration.

## Additional file

Additional file 1:
**The method described in this paper was used to determine volume and axis orientation in two patients.** Computer models of the pelvis were created based on preoperative evaluative CT scans in Case 1, a 13-year-old boy with cerebral palsy and left hip joint dislocation **(Figure S1a)**, and Case 2, a 13-year-old girl with cerebral palsy and right hip joint dislocation **(Figure S2a)**. The results are summarized in **Table S1** and depicted **Figures S1b and S2b**. Comparison of measurements of normal and pathologic acetabula in these patients shows large differences in acetabular orientation in the spastic hip. The acetabular axis determined using our method has a completely reversed orientation under dysplastic conditions (retroversion, posterior tilt, inclination over 90 degrees), which may influence decisions regarding surgical redirection of the acetabulum. These cases demonstrate the applicability of our method in clinical treatment. There are also differences between both dysplastic acetabula with regard to volume, a result that does not support the common view of a shallow, small-volume dysplastic acetabulum. It should be noted, however, that these are only two cases and that further investigation is warranted. **Figure S1.** Standard X-ray and CT reconstruction in case 1, a 13-year-old boy with cerebral palsy and left hip joint dislocation: (a) Pelvic anteroposterior X-ray; (b) pelvic 3D CT reconstruction showing the position of the acetabular axis. 3D, three-dimensional; CT, computed tomography. **Figure S2.** Standard X-ray and CT reconstruction in case 2, a 13-year-old girl with cerebral palsy and right hip joint dislocation: (a) Pelvic anteroposterior X-ray; (b) 3D CT reconstruction showing the position of the acetabular axis. 3D, three-dimensional; CT, computed tomography. **Table S1.** Surface, volume, and spatial orientation of the acetabulum.

## Abbreviations

2D: Two-dimensional
3D: Three-dimensional
AAA: Acetabular anteversion angle
AAI: Anterior acetabular index
APP: Anterior pelvic plane
ASIS: Anterior superior iliac spine
SB: Base of the sacrum
CT: Computed tomography
ICCs: Intraclass correlation coefficients
ISB: International society of biomechanics
MRI: Magnetic resonance imaging
PSIS: Posterior superior iliac spine
STC: Standardization and terminology committee of the international society of biomechanics
