# Supplementary material for: An accurate method of radiological assessment of acetabular volume and orientation in computed tomography spatial reconstruction
Source: BMC Musculoskelet Disord. 2015 Feb 25;16:42. doi: 10.1186/s12891-015-0503-8 (PMC4351831; doi:10.1186/s12891-015-0503-8)
Supplement: Additional file 1: — The method described in this paper was used to determine volume and axis orientation in two patients. Computer models of the pelvis were created based on preoperative evaluative CT scans in Case 1, a 13-year-old boy with cerebral palsy and left hip joint dislocation (Figure S1a), and Case 2, a 13-year-old girl with cerebral palsy and right hip joint dislocation (Figure S2a). The results are summarized in Table S1 and depicted Figures S1b and S2b. Comparison of measurements of normal and pathologic acetabula in these patients shows large differences in acetabular orientation in the spastic hip. The acetabular axis determined using our method has a completely reversed orientation under dysplastic conditions (retroversion, posterior tilt, inclination over 90 degrees), which may influence decisions regarding surgical redirection of the acetabulum. These cases demonstrate the applicability of our method in clinical treatment. There are also differences between both dysplastic acetabula with regard to volume, a result that does not support the common view of a shallow, small-volume dysplastic acetabulum. It should be noted, however, that these are only two cases and that further investigation is warranted. Figure S1. Standard X-ray and CT reconstruction in case 1, a 13-year-old boy with cerebral palsy and left hip joint dislocation: (a) Pelvic anteroposterior X-ray; (b) pelvic 3D CT reconstruction showing the position of the acetabular axis. 3D, three-dimensional; CT, computed tomography. Figure S2. Standard X-ray and CT reconstruction in case 2, a 13-year-old girl with cerebral palsy and right hip joint dislocation: (a) Pelvic anteroposterior X-ray; (b) 3D CT reconstruction showing the position of the acetabular axis. 3D, three-dimensional; CT, computed tomography. Table S1. Surface, volume, and spatial orientation of the acetabulum. [file 12891_2015_503_MOESM1_ESM.docx]

**Additional file 1**

The method described in this paper was used to determine volume and axis orientation in two patients. Computer models of the pelvis were created based on preoperative evaluative CT scans in Case 1, a 13-year-old boy with cerebral palsy and left hip joint dislocation (Figure A1a), and Case 2, a 13-year-old girl with cerebral palsy and right hip joint dislocation (Figure A2a). The results are summarized in Table A1 and depicted Figures A1b and A2b. Comparison of measurements of normal and pathologic acetabula in these patients shows large differences in acetabular orientation in the spastic hip. The acetabular axis determined using our method has a completely reversed orientation under dysplastic conditions (retroversion, posterior tilt, inclination over 90 degrees), which may influence decisions regarding surgical redirection of the acetabulum.

These cases demonstrate the applicability of our method in clinical treatment. There are also differences between both dysplastic acetabula with regard to volume, a result that does not support the common view of a shallow, small-volume dysplastic acetabulum. It should be noted, however, that these are only two cases and that further investigation is warranted.

**Figure Legends**

**Figure A1** **Standard X-ray and CT reconstruction in case 1, a 13-year-old boy with cerebral palsy and left hip joint dislocation:** (a) Pelvic anteroposterior X-ray; (b) pelvic 3D CT reconstruction showing the position of the acetabular axis. 3D, three-dimensional; CT, computed tomography.

**
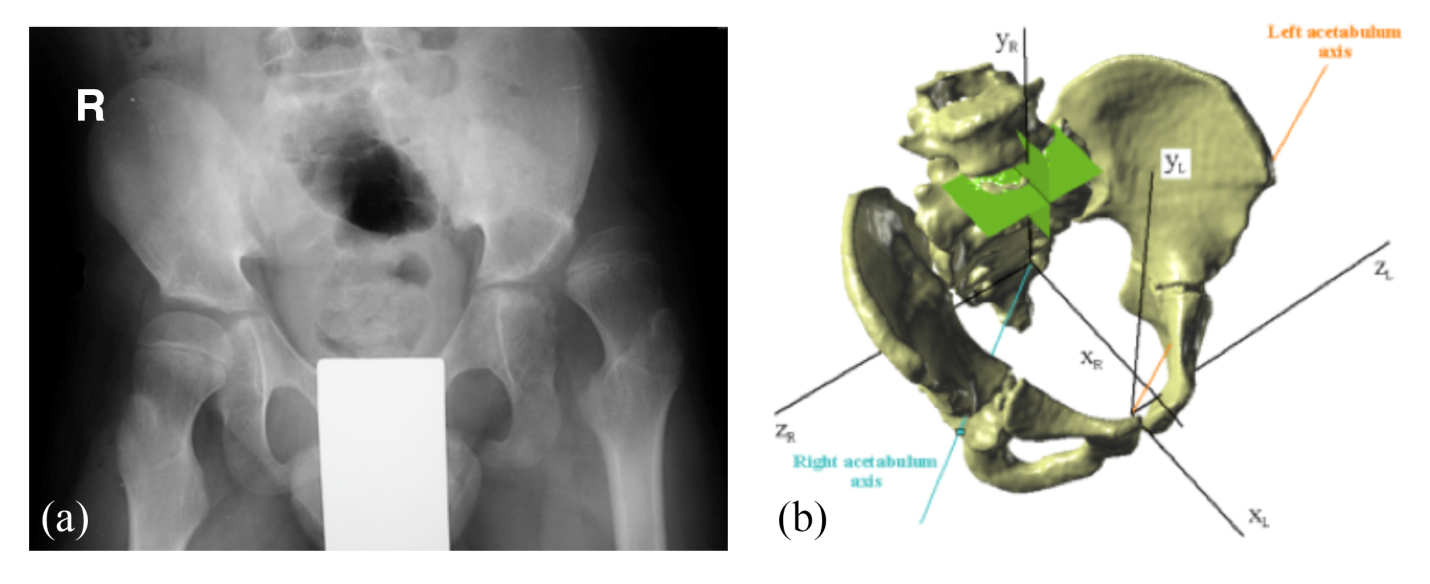
**

**Figure A2 Standard X-ray and CT reconstruction in case 2, a 13-year-old girl with cerebral palsy and right hip joint dislocation:** (a) Pelvic anteroposterior X-ray; (b) 3D CT reconstruction showing the position of the acetabular axis. 3D, three-dimensional; CT, computed tomography.


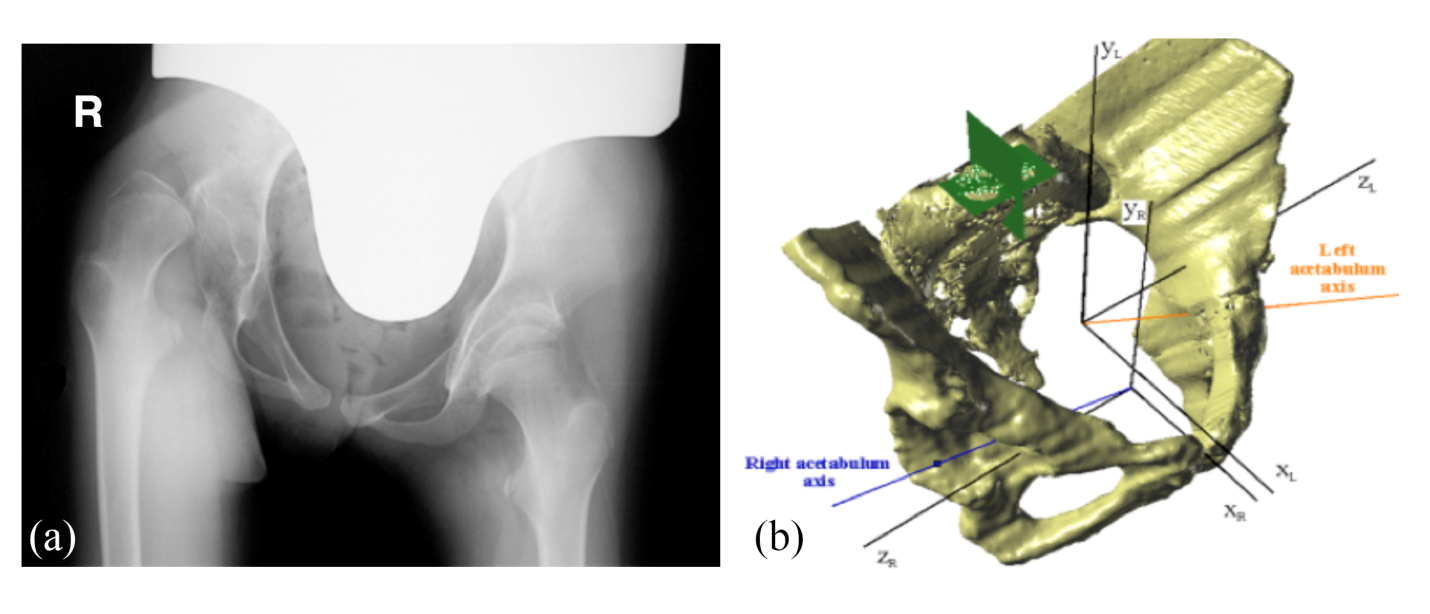


**Table A1 Surface, volume, and spatial orientation of the acetabulum**

|  | | **Volume**  **(mL)** | **Inclination angle (degrees)** | **Anteversion angle (degrees)** | **Tilt angle (degrees)** |
| --- | --- | --- | --- | --- | --- |
| **Case 1^a^** | **Right** | 24.2 | 75.3 | 30.5 | 24.1 |
|  | **Left*** | 22.7 | 99.9 | –22.5 | –157.2 |
| **Case 2^b^** | **Right*** | 27.3 | 98 | –6.1 | –126.9 |
|  | **Left** | 26.9 | 70.6 | 16 | 50.8 |

*Affected side

^a^13-year-old male with cerebral palsy and left hip joint dislocation.

^b^13-year-old female with cerebral palsy and right hip joint dislocation.
